# Automation in vitrification and thawing of mouse oocytes and embryos

**DOI:** 10.3389/fcell.2023.1330684

**Published:** 2023-12-21

**Authors:** Yan Zhu, Quan-Jun Zhang, Huai L. Feng, Jin Luo, Shu Miao, Man-Xi Jiang

**Affiliations:** ^1^ Guangdong Second Provincial General Hospital, Guangzhou, China; ^2^ Institutes of Biomedicine and Health, Chinese Academy of Sciences, Guangzhou, China; ^3^ New York Fertility Center, New York-Prebyterian Healthcare System Affiliate Weill Cornell Medical College, New York, NY, United States; ^4^ T Stone Robotics Institute, Department of Mechanical and Automation Engineering, The Chinese University of Hong Kong, Shenzen, China; ^5^ Department of Automation, Tsinghua University, Beijing, China

**Keywords:** cryo-handle, automated vitrification and thawing, oocytes, embryos, mice

## Abstract

Vitrification is a common technique for cryopreserving oocytes or embryos. However, manual vitrification is tedious and labor-intensive, and can be subject to variations caused by human factors. To address these challenges, we developed an automated vitrification-thawing system (AVTS) based on a cryo-handle. Our study firstly assessed the efficiency of cryoprotectant exchange through comparing the osmolalities of fresh and collected solutions during automated vitrification and thawing, and evaluated the cooling and warming rates of the cryo-handle. We also compared mouse oocyte survival, fertilization and embryo development after thawing and ICSI, and the development of re-frozen cleavage embryos between manual operation and automated system. The results showed that the osmolalities of collected samples were within normal range and comparable to fresh solutions. Furthermore, the automated system could obtain the reliable cooling and warming rates. Particularly, there were no significant differences in oocyte survival rates, fertilization rates, and subsequent embryo development and its quality between two procedures. Our findings suggest that AVTS has no impact on osmolalities of vitrification and thawing solutions, ensuring the proper exchange of cryoprotectants. The cryo-handle also shows the ability to achieve reliable cooling and warming rates, which benefits for the cryopreservation and thawing process. Moreover, the results from mouse oocytes and embryos indicate that automated system has effectively maintained the survival and fertilization of frozen oocytes and supported subsequent embryo development. Therefore, the automated vitrification and thawing system will inevitably represent a superior alternative to manual operation.

## Introduction

Cryopreservation of oocytes and embryos by means of vitrification is a crucial part of assisted reproductive technology (ART), as it not only maximizes pregnancy rates from a single oocyte collection cycle but also reduces the risk of ovarian hyperstimulation syndrome (OHSS), ensuring patient health and safety. Clinical outcomes have shown high rates of oocyte/embryo recovery, successful pregnancies, live births, and neonatal health (Kuwayama et al., 2005; Takahashi et al., 2005; Kato et al., 2012). However, the development of vitrification techniques was overshadowed by slow freezing for a long time until the first successful vitrification of mouse embryos was achieved in 1985 (Rall and Fahy, 1985).

During vitrification, the rapid cooling and warming process helped to avoid chilling injury for oocytes and the high viscosity of the cryoprotectants in the vitrification solution prevented the formation of ice crystals (Saragusty and Arav, 2011; Arav, 2014). Currently, vitrification is becoming the preferred method for preserving oocytes and embryos, and it consistently yields highly satisfactory outcomes (Cobo et al., 2013; Levi-Setti et al., 2016). Nevertheless, the existence of various vitrification protocols and cryo-carriers, and the differences in personnel operation complicate the reproducibility of this technology (Roy et al., 2014a). In particular, the manual process of vitrification involves numerous variables and demands a high level of skill, resulting in inconsistent outcomes between operators and ART clinics (Gosden, 2011). Thus, a standardized automated operation is necessary for vitrification. Automated vitrification has the potential to provide a more consistent and repeatable process, enabling all operators to achieve stable outcomes. Recently, semi-automated Shara and automated Gavi systems have been developed to vitrify oocytes and embryos, yielding equivalent *in vitro* outcomes in animals and humans, and presenting a significant potential for clinical application (Roy et al., 2014b; Roy et al., 2017; Arav et al., 2018; Brunetti et al., 2021).

In our present program, a new type of carrier named as cryo-handle, and a programmable automated system were developed to perform vitrification and thawing. In order to verify the exchange efficiency of cryoprotective solutions including all kinds of vitrification solutions (VS) and thawing solutions (TS), the automated vitrification and thawing system (AVTS) was firstly run in empty mode, in which the cryo-handle did not include oocytes or embryos; the samples of VS and TS solution in cryo-handles were collected for osmolality determination, and the fresh solutions were the controls. The next, the cooling and warming rates of cryo-handle were detected by using a mock experiment. Additionally, mouse oocytes and embryos were cryopreserved by AVTS and manual vitrification. Finally, oocyte survival rates, fertilization rates, and embryo development after thawing were assessed between AVTS and manual vitrification/thawing groups, and the development of blastocysts from refrozen cleavage embryos was compared between two groups.

## Materials and methods

### Humane care and use of animals

Female and male B6 mice, aged 8 and 10 weeks respectively, were utilized for the experiments. The mice were bred and maintained at a controlled temperature of approximately 22°C with a 14-h light and 10-h dark cycle at the Animal Center of Guangdong Second Provincial General Hospital. All experimental protocols were performed after obtaining the approval from the ethical committee of Guangdong Second Provincial General Hospital for animal experiments (No. 2014-KYLL-015) and all the used methods are reported in accordance with the Animal Research Reporting of in vivo Experiments (ARRIVE) guidelines. Euthanasia was performed by CO_2_ asphyxiation followed by cervical dislocation according to American Veterinary Medical Association (AVMA) guidelines.

### Description of the automated vitrification and thawing system (AVTS) and cryo-handle

**FIGURE 1:**
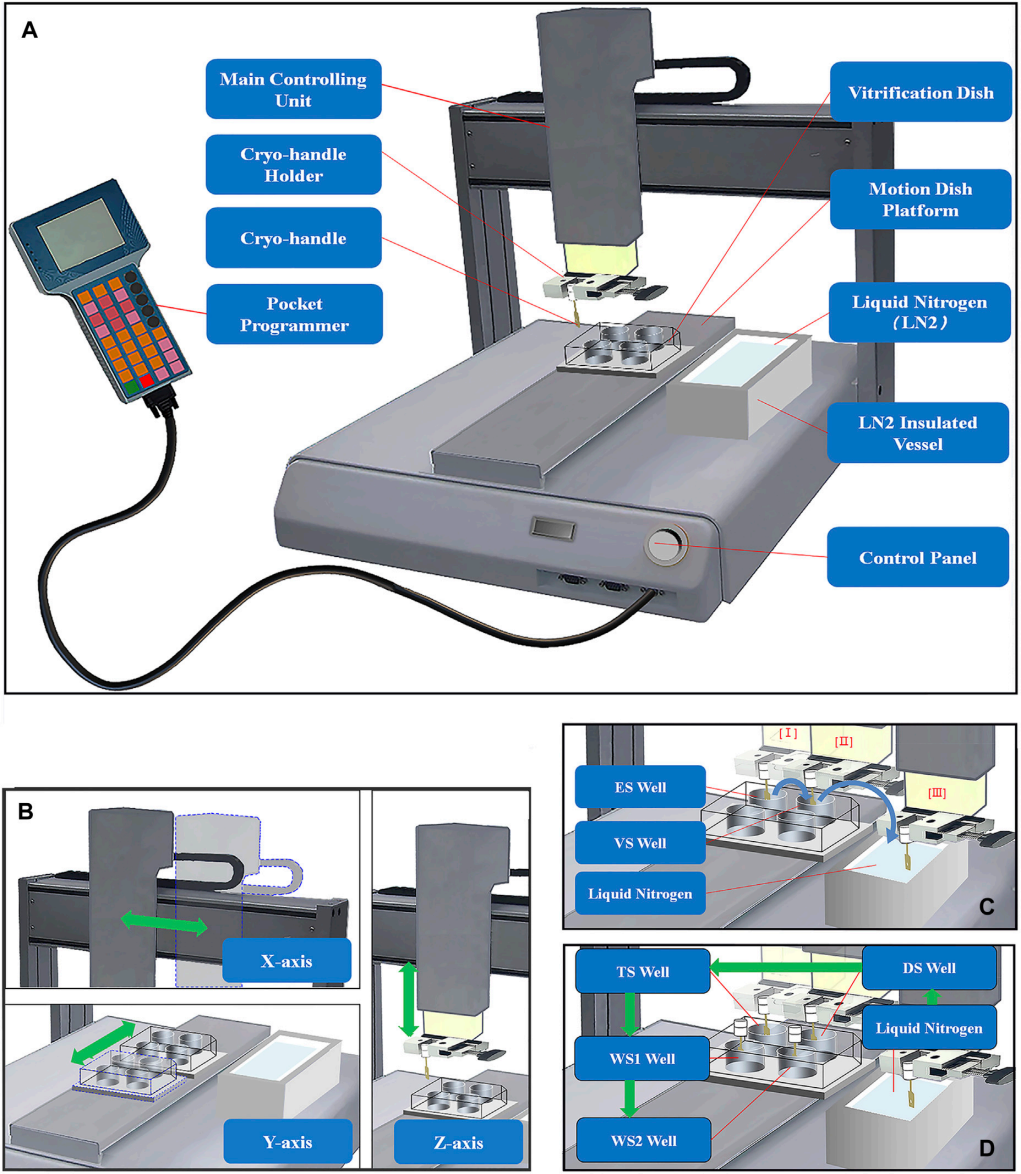
Structure Diagram of Automated Vitrification and Thawing System (AVTS). **(A)** The automated vitrification and thawing system mainly consists of a main controlling unit, a motion dish platform, a pocket programmer, and an LN insulated vessel; **(B)** The main controlling unit and vitrification dishes move in the x-, y-, and z-axial directions, and the green double-headed arrows represent the directions of movements; **(C)** The full process of automated vitrification includes equilibration in ES solution (I), exposure in VS solution (II), and a final holding step in LN of an insulated vessel, and the directions of the blue curved arrows indicate the sequential steps of vitrification; **(D)** Before thawing, it is necessary to briefly immerse the cryo-handle in LN firstly, and then the frozen bio-samples are sequentially exposed to TS, DS, WS1 and WS2 solutions at specific intervals during automated thawing. The green arrows indicate the sequential steps of the thawing process.

The automated vitrification and thawing system is comprised of a vertical main controlling unit equipped with a carrier holder, a motion dish platform, a pocket programmer (Figure 1A, Jiaheng Intelligent Equipment Co. Shenzhen, China), and a liquid nitrogen (LN) insulated vessel. The main controlling unit is responsible for controlling the motion space and the pre-set positions based on initial coordinates and time intervals of different actions in the x- and z-axial directions (up-left panel and right of Figure 1B). The platform is used to fix the VS or TS dishes in place and control their movement in the y-axial direction (low-left panel of Figure 1B). The accuracy of the position control is achieved through the proportional, integral, and derivative (PID) control algorithm (Chang et al., 2016; Zhu et al., 2023). The pocket programmer is used to pre-program the given positions and time intervals, which are responsible for controlling the orderly exposure of bio-samples in the cryo-carriers to different VS (Figure 1C) or TS solutions (Figure 1D) at specific time intervals.

**FIGURE 2:**
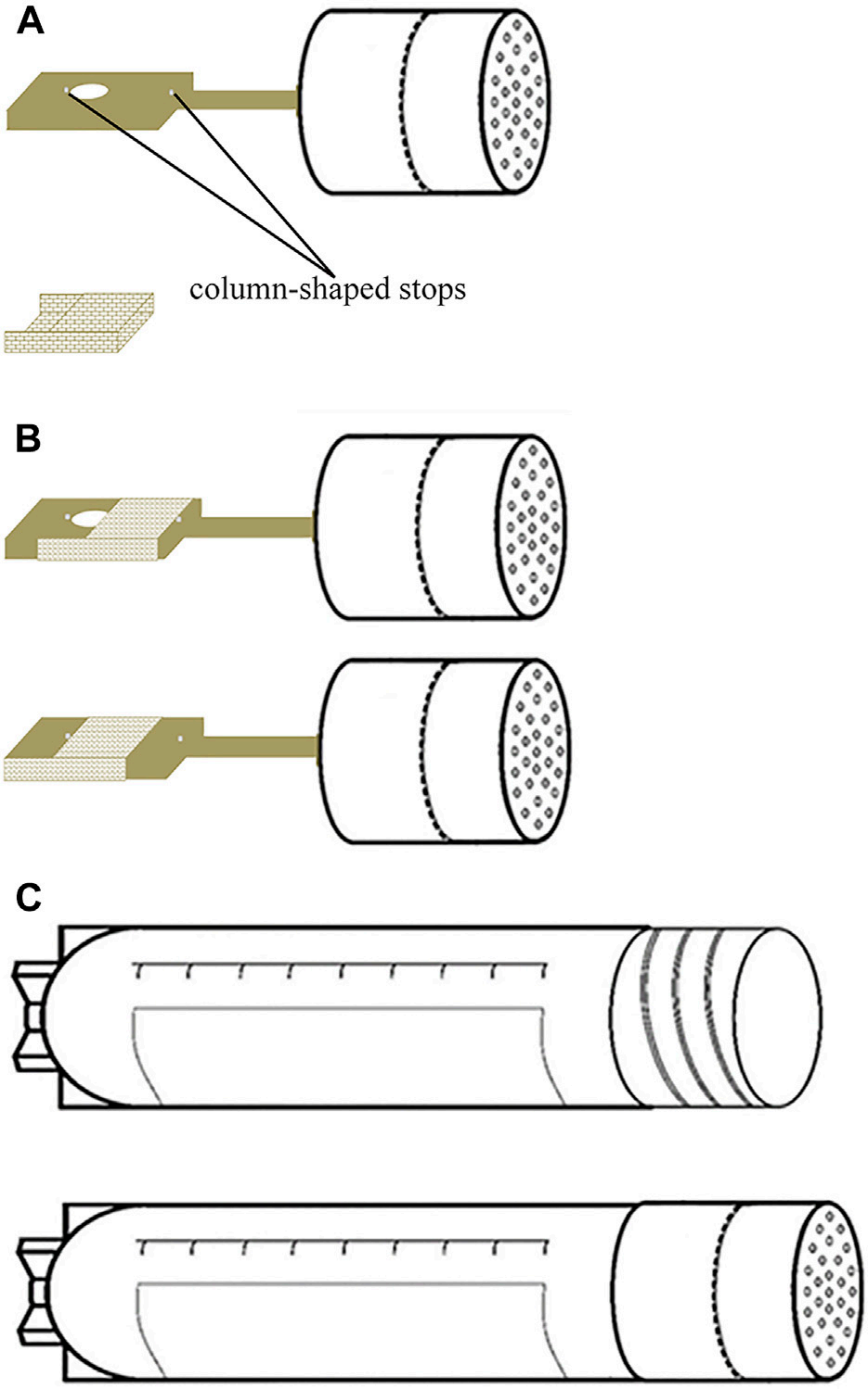
Structure Diagram of Cryo-handle. **(A)** The cryo-handle consists of a rectangular copper handle with a round cavity (hole) installed two miniature column-shaped stops on both sides of the hole (upper panel), and a drawer-style mesh-like copper casing (lower panel); **(B)** the cryo-handle before sample loading (upper panel) and the cryo-handle which round hole is covered by a mesh-like casing after sample loading (lower panel); **(C)** the protective cryo-tube (upper panel) and the cryo-handle sealed with a protective cryo-tube (lower panel).

The structure and usage of the cryo-handle are depicted in the schematic diagram in Figure 2. The cryo-handle consists of a rectangular copper handle with a round hole (upper panel of Figure 2A), a drawer-style mesh-like copper casing (lower panel of Figure 2A), and a protective cryo-tube (upper panel of Figure 2C). Inside the cryo-handle, there is a round hole designed to accommodate bio-samples. Additionally, miniature column-shaped stops are present on both sides of the cryo-handle, serving to prevent the outer casing from falling off during pulling and pushing (Figure 2A). The rectangular handle measures 10 mm in length, 2 mm in width, and 1 mm in height. The round hole has a radius of 0.5 mm and a height of 1 mm.

To use the cryo-handle, it is recommended the mesh-like casing is first pushed towards the far right of the cryo-handle (upper panel of Figure 2B). Then, the bio-samples are placed into the hole (cavity) within the holding medium (HM). Afterward, the casing is promptly pushed towards the far left (lower panel of Figure 2B), ensuring that it securely sits in the HM for subsequent freezing procedures.

### Measurement of osmolalities of vitrification and thawing solutions

To confirm whether complete exchange had taken place between different solutions during the vitrification and thawing procedures, we did a simulation testing by using the empty cryo-handle. By comparing the osmolalities of the collected samples from cryo-handles with the corresponding fresh solutions, we were able to evaluate the exchange efficiency of different solutions within the cryo-handle during the simulated vitrification and thawing processes.

Equilibrium (ES), thawing (TS), dilution (DS), washing (WS1), and WS2 solutions were collected in volumes of at least 30 μL, and their osmolalities were directly measured at least three times using an osmometer (OSMOMAT030, Cryoscopic Osmometer, Germany). However, the osmolality of the VS solution exceeded the measurement range of the osmometer, which is limited to 0–3,000 mOsm/Kg. Therefore, we planned to use the fitting curve method to indirectly determine the osmolality of the VS solution.

To determine the osmolality of both fresh and the collected VS solutions, the VS solution and the collected sample were initially diluted by using WS2 at ratios of 1/20, 1/15, 1/10, and 1/5. Subsequently, the osmolalities of diluted solutions were measured using the Osmometer. Then, the fitting curves of fresh and the collected VS solutions we obtained by using GraphPad Prism 8.0 software (GraphPad Software Inc., La Jolla, CA, United States), and finally these fitting curves were used to calculate the original osmolalities of fresh and the collected VS solutions. Each solution needs to be fitted three times in order to obtain the original osmolalities of both the fresh and collected VS solutions.

### Measurements of the cooling and warming rates of cryo-handle

**FIGURE 3:**
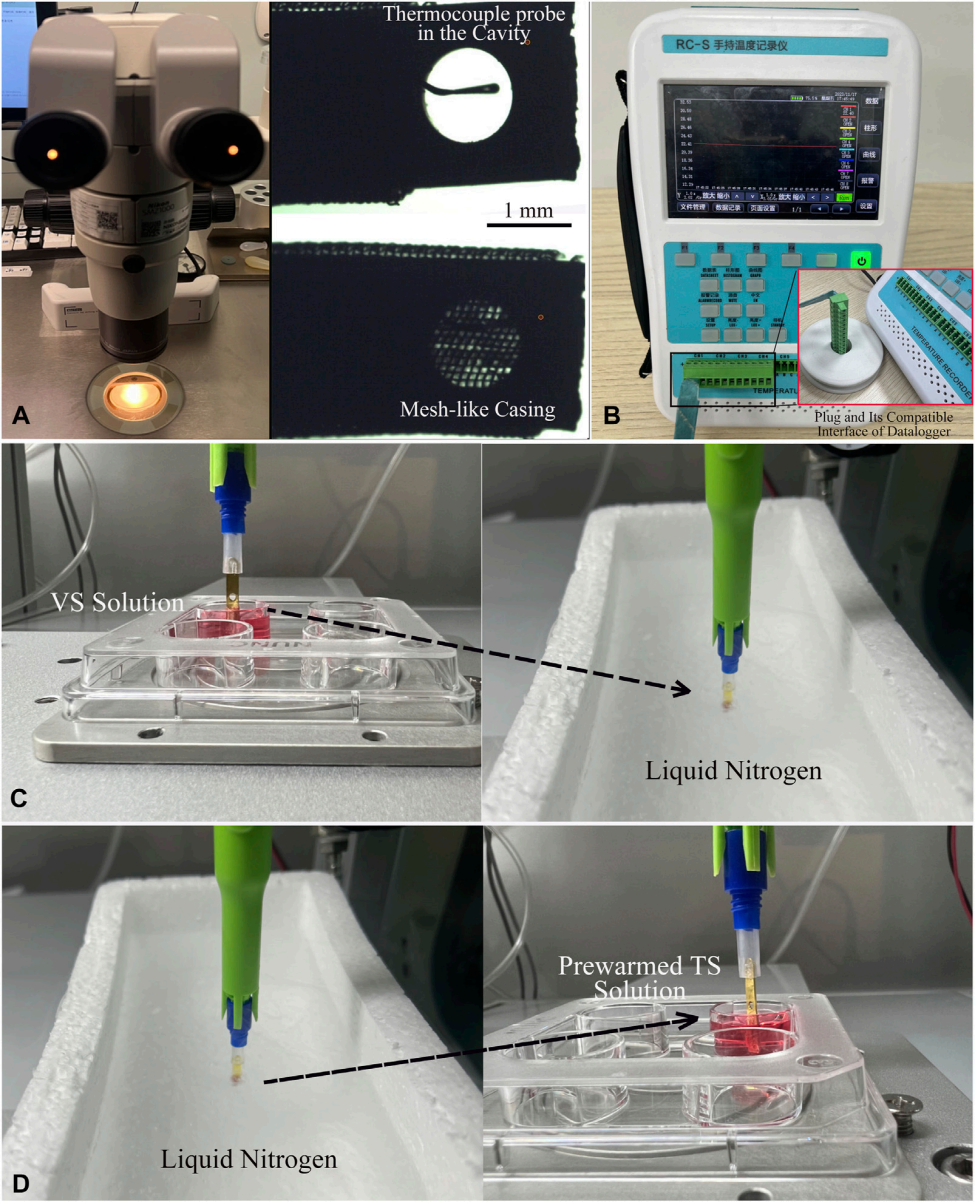
Process of Thermocouple Assembly and *In-situ* Temperature Measurement of Cryo-handle. **(A)** Under the 15X stereo microscope, a T-type thermocouple probe (φ0.02 mm) was inserted into the central point of the cryo-handle’s cavity. Then, 1.5 μL of VS solution was added into this cavity, and finally, the mesh-like casing was moved to cover the cavity completely. **(B)** The plug at the end of the thermocouple’s wire was inserted into the compatible interface of the data logger. **(C)** After activating the data logger to initiate the temperature measurement, the cryo-handle with the thermocouple probe was immersed into liquid nitrogen and held in liquid nitrogen for a while. **(D)** The cryo-handle was then taken out from the liquid nitrogen and placed into a well containing the pre-warmed TS solution. The temperature at each time point was recorded *in situ* during the whole process. The dashed arrows indicated the movement direction of the cryo-handles.

The thermocouple (TC) assembly was executed under a 15X stereo microscope (Olympus SMZ, Japan). Initially, a thin T-type thermocouple probe (φ0.02 mm) was carefully inserted into the central point of the cryo-handle’s cavity. Following this, a precise volume of 1.5 μL of VS solution was swiftly added to the cavity. To secure the probe at the desired measurement point, a mesh-like casing was then pushed into place, covering the cavity effectively (Figure 3A). Finally, the plug of the thermocouple’s fine wire was connected to the compatible interface of the datalogger (RC3008S, RUIBEST Electronics Co., Ltd. Changzhou, China). The datalogger was activated to initiate the temperature measurement, enabling continuous monitoring of the readings at intervals of 100 m (Figure 3B). The temperature at each time point was recorded *in situ* when the cryo-handle was immersed into LN or transferred from LN into a well of a 4-well-dish containing 1.0 mL of TS solution pre-warmed to 37°C. Upon completion of the measurement procedure, all temperature data in CSV format were transferred to a laptop computer for data analysis. Microsoft Excel was employed to analyze the temperature trend in this study. The formula used to calculate the cooling (CR) and warming (WR) rates (°C/minute) is [ΔT (°C)/Δt (ms)] × 1,000 × 60. The calculation of the cooling and warming rates was conducted at least three times.

### Collection of MII oocytes

Female B6 mice were administered intraperitoneal injections of 7.5 IU of pregnant mare serum gonadotrophin (PMSG) from Ningbo Sansheng Hormone Factory, Ningbo, China. This was followed by intraperitoneal injection of 7.5 IU of human chorionic gonadotrophin (hCG) also from Ningbo Sansheng, 48 h later. After 13–14 h of the hCG injection, the mice were euthanized by cervical dislocation. The oviducts were then removed and placed in 1 mL of M2 medium from Sigma-Aldrich Chemical Co., St. Louis, MO. To release cumulus oocyte complexes (COCs), the ampullae of the oviducts were torn. The cumulus cells were then enzymatically removed using 80 IU/mL of hyaluronidase from Sigma-Aldrich, followed by mechanical dissociation using a glass pipette. Only morphologically normal mature metaphase II (MII) oocytes, identified by the presence of a first polar body, were selected for further use.

## Vitrification and thawing of MII oocytes

### Manual procedure

Following the manufacturer’s instructions (Kitazato Co., Minato-ku, Japan) with slight modifications (Kuwayama et al., 2005; Castelló et al., 2018), four to six MII oocytes were initially suspended in 500 μL of ES for 10 min. They were then transferred to 500 μL of VS for 90 s at room temperature. Subsequently, the oocytes from VS were loaded onto the tip of the Cryotop (Kitazato Co.) with a volume of 2–3 μL and immediately plunged into LN for storage. After vitrification, the Cryotop should be sealed with a protective cap under LN to prevent exposure of the vitrified oocytes to room temperature.

Oocytes were thawed by immersing the Cryotop directly into 500 μL of pre-warmed TS solution at 37°C for 1 min after removing the cap under LN. The thawed oocytes were then transferred to DS for 3 min, washed twice with WS1 and WS2 for 5 min each, and finally transferred to HTF medium (Millipore Co., Billerica, MA) and incubated at 37°C in 5% CO_2_ in a humidified air for 2 h.

### AVTS procedure

First, the VS dish containing 800 μL of holding medium (HM), ES, and VS solutions in each well was placed onto the appropriate position of the motion platform. Next, four to six MII oocytes were transferred from HTF medium into the cavity of a cryo-handle containing HM in a central-well dish, using a mouth-controlled pipette under a stereo microscope (Olympus SMZ, Japan) at 37°C.The cryo-handle was immediately enclosed with a drawer-style mesh-like casing (Figure 2B) and fixed onto the carrier holder of the main controlling arm (Figure 1A). The system was switched on to initiate the vitrification procedure. As per the pre-established AVTS program, the Cryo-handle was exposed in an orderly manner to 800 μL of ES for 10 min and 800 μL of VS for 90 s in a rotating stirred way. Once all the preparatory steps were completed, the Cryo-handle was swiftly taken off from the robotic arm and sealed by a cryotube in LN (refer to Figure 1C). Finally, the sealed Cryo-handle was stored in LN tanks for long-term preservation.

Before thawing, a 4-well-dish with 800 μL of TS (prewarmed to 37°C), DS, WS1, and WS2 in each well was prepared and placed onto the dish platform. The Cryo-handle containing oocytes was carefully removed from the protective tube and securely attached to the robotic arm, ensuring that the tip of the Cryo-handle remained immersed in liquid nitrogen. The system was then activated to commence the thawing procedure. Following the automated thawing protocol showed as Figure 1D, the Cryo-handle with the frozen bio-samples was firstly installed on the cryo-carrier holder, and then in a rotating stirred manner methodically exposed to 800 μL of TS (prewarmed to 37°C) for 60 s and 800 μL of DS for 3 min, followed by 800 μL of WS1 and WS2 for 5 min each. Subsequently, the Cryo-handle was disconnected from the robotic arm, allowing the oocytes to be released into HTF medium. The oocytes were then incubated at 37°C in a 5% CO_2_ environment with humidified air for 2 h.

The survival of thawed oocytes was evaluated through morphological analysis, which involved examining the integrity of the plasma membrane and the appearance of the ooplasm for any discoloration. The survival oocytes were subsequently incubated at 37°C in a 5% CO_2_ environment with humidified air until the performance of intracytoplasmic sperm injection (ICSI).

### Intracytoplasmic spermatozoa injection (ICSI)

Epididymal spermatozoa were obtained from the cauda epididymis of 8- to 10-week-old B6 mice and subsequently incubated in HTF medium for 30 min at 37°C in an environment containing 5% CO_2_. Afterwards, 1 µL of the spermatozoa suspension was combined with 10 µL of 10% polyvinyl pyrrolidone (PVP)-HEPES-buffered CZB medium in an ICSI manipulation chamber. The ICSI procedure was carried out following previously described methods (Jiang et al., 2005) with a minor modification. Briefly, the sperm head was isolated from the tail using piezo pulses targeting the neck region. The separated head was promptly injected into a thawed oocyte. Following a 10-min recovery period at room temperature, the oocytes were thoroughly washed at least three times before being transferred to KSOMaa medium (potassium simplex optimized medium with amino acid, Millipore Co.). Fertilization success was determined by the presence of two pronuclei (2 PN) in the zygote, observed 5 h after the ICSI procedure. The developmental progression of the embryos to the cleavage stage was assessed on day 2.5 post-insemination.

### Vitrification and thawing of cleavage embryos

All ∼ 8-cell stage embryos, obtained from both manually and AVTS vitrified oocytes, were vitrified and thawed using the respective manual and AVTS protocols. The viability of these embryos was assessed by evaluating their ability to resume development and by determining the rates of blastocyst formation and hatching.

### Manual procedure

Three to four 8-cell embryos were initially suspended in 500 μL of ES for 7 min, followed by transfer to 500 μL of VS for 45–60 s at room temperature. The embryos, with approximately 2–3 μL of VS, were then loaded onto the tip of the Cryotop and promptly submerged into LN. Finally, the Cryotop was sealed with a protective cap in LN for long-term storage.

During thawing, after removing the cap in LN, the embryos were thawed by immersing the tip of the Cryotop directly into 500 μL of TS solution pre-warmed to 37°C for 1 min. Subsequently, the embryos were transferred to 500 μL of DS for 3 min, followed by two washes with 500 μL of WS1 and WS2 for 5 min each. Finally, the embryos were transferred to KSOMaa medium (Millipore Co., Billerica, MA) and incubated at 37°C in 5% CO_2_ in humidified air until Day 3.5.

### AVTS procedure

After the Cryo-handle containing three to four embryos was placed onto the robotic arm. The system was then activated to initiate the vitrification procedure. During AVTS, the cryo-handle was successively exposed to 800 μL of ES for 7 min and 800 μL of VS for 60 s, using a rotating stirred method. Once the entire process was completed, the Cryo-handle was swiftly detached from the robotic arm and carefully inserted into a cryo-tube for sealing in LN. Finally, the tightly sealed Cryo-handle was stored in LN tanks for long-term preservation.

The Cryo-handle containing embryos was disconnected from the cryotube and placed on the robotic arm before thawing, ensuring that the tip of the Cryo-handle remained immersed in liquid nitrogen in the entire process. The system was then activated to initiate the thawing procedure. The cryo-handle was sequentially exposed to 800 μL of TS (prewarmed to 37°C) for 60 s, 800 μL of DS for 3 min, 800 μL of WS1 for 5 min, and 800 μL of WS2 for 5 min, using a rotating stirred method. Finally, after disconnecting the Cryo-handle from the robotic arm, the embryos were released into KSOMaa medium and incubated at 37°C in 5% CO_2_ in humidified air until Day 3.5.

### Statistical analysis

The data were analyzed using GraphPad Prism 8 software (GraphPad Software Inc., San Diego, CA, United States). The fitting curve of osmolalities were calculated using single linear regression, and osmolality comparisons of VS and TS solutions were made using ordinary one-way ANOVA. The rates of survival oocytes and fertilization, and the percentages of 8-cell cleavage embryos, blastocysts and hatching blastocysts were also compared using ordinary one-way ANOVA. Statistical significance was defined as a *p*-value of less than 0.05.

## Results

### Fitting curves for osmolality of the VS solutions and changes in osmolality of fresh or collected vitrification/thawing solutions

**FIGURE 4:**
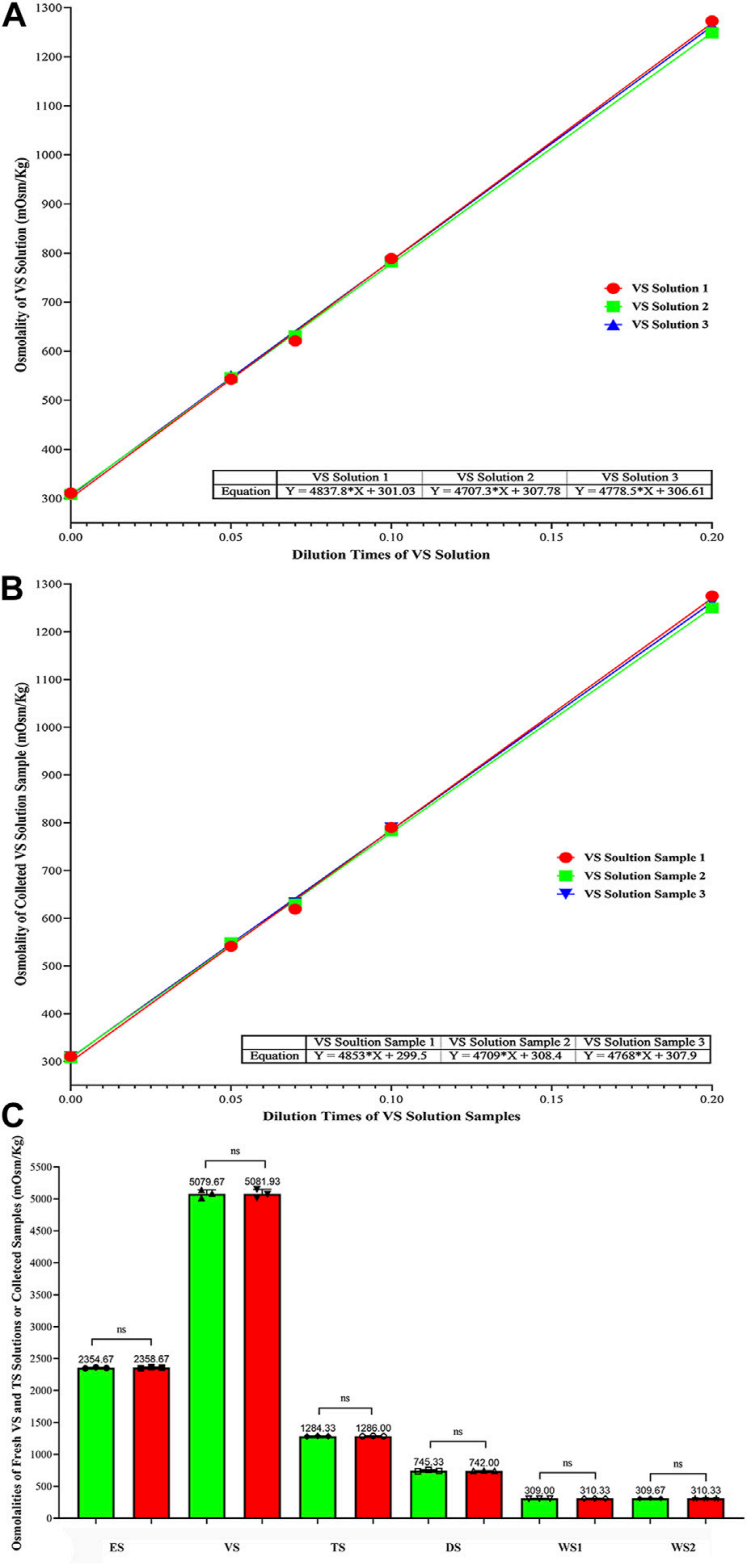
Comparison of Osmolalities Between Fresh and the Collected VS/TS solutions, and Osmolality Change Trend of all Kinds of solutions (mOsm/Kg). **(A)** Fitting curve equations of osmolalities for VS solutions; **(B)** Fitting curve equations of osmolalities for collected VS samples; **(C)** Comparison of osmolalities (mean ± SD) between fresh and the collected solutions. Data were analyzed by ordinary on-way ANOWA; NS represents no significance.

Through the simple linear regression analysis by using GraphPad Prism 8.0 (Supplementary Tables S1, S2), the osmolality fitting equations of fresh VS solution and the collected VS samples were obtained as follows:

Y_VS1_ = 4,837.8X + 301.03, Y_VS2_ = 4,707.3X + 307.78, Y_VS3_ = 4,778.5X + 306.61 and Y_Sample1_ = 4,853X + 299.5, Y_Sample2_ = 4,709X + 308.4, Y_Sample3_ = 4,768X + 307.9, respectively (Supplementary Tables S1, S2); The average osmolalities of fresh and collected VS solutions ((standard deviations (SD)) when X equals to 1.0 were 5,079.67 ± 62.05 and 5,081.93 ± 67.75 mOsm/Kg, respectively (Figures 4A,B; Supplementary Table S1, Supplementary Table S2 and Supplementary Table S3).

The osmolalities of fresh and the collected solutions (mean ± SD) were compared, and no significant differences were found (Figure 4C and Supplementary Table S3). The osmolalities of ES, VS, TS, DS, WS1 and WS2 between fresh and the collected samples were 2,354.67 ± 8.33 vs*.* 2,358.67 ± 10.07, 5,079.67 ± 62.05 vs*.* 5,081.93 ± 67.75, 1,284.33 ± 3.51 vs*.* 1,286.00 ± 3.46, 745.33 ± 12.86 vs*.* 742.00 ± 3.46, 309.00 ± 1.00 vs*.* 310.33 ± 1.15, and 309.67 ± 1.53 vs*.* 310.33 ± 0.58 mOsm/Kg, respectively (*p* > 0.999). This indicates that the osmolality of solutions collected from cryo-handle did not show significant changes compared to fresh solutions. The trend of osmolalities was almost identical between the two groups, suggesting that the VS and TS solutions were fully exchanged during automated vitrification and thawing.

### Temperature dynamics of the cryo-handle during vitrification and thawing

**FIGURE 5:**
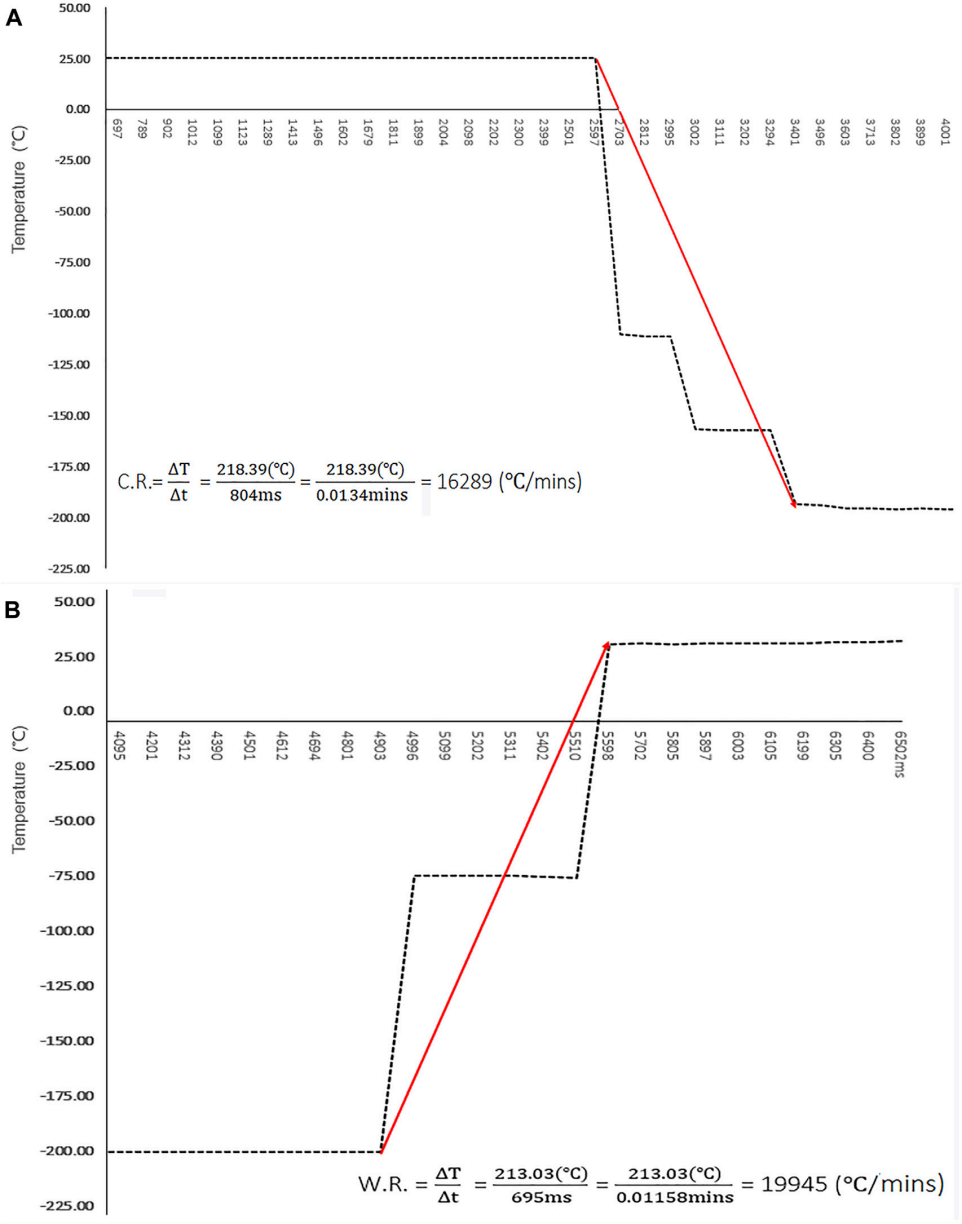
Temperature Dynamics of Cryo-handle During Vitrification and Thawing. **(A)** The temperature in the round hole of cryo-handle during the vitrification procedure was measured using a digital datalogger, and the results were plotted as a curve graph. The temperature changes are shown at intervals of approximate 100 m, starting from immersion the cryo-handle into Liquid Nitrogen. The cooling rate is represented by the slope between the highest and lowest temperature points. Red arrow indicates the trend of temperature changes; **(B)** The temperature in the hole of cryo-handle during the warming procedure was measured using a digital datalogger, and the results were plotted as a curve graph. The temperature changes are shown at intervals of approximate 100 m. The warming rate is represented by the slope between the highest and lowest temperature points. Red arrow indicates the trend of temperature changes.

The temperature dynamics of the cryo-handle were estimated using a bio-sample free simulation test (as shown in Figure 5A and Supplementary Table S4). The temperature at the hole of cryo-handle decreased from 24.99°C to −193.40°C within nearly 0.80s and remained sufficiently cool after immersion in LN. The cooling rates reached up to 16, 298°C/min.

The dynamics observed during the warming procedure are illustrated in Figure 5B and Supplementary Table S4. In the cryo-handle condition, it was observed that the temperature rapidly increased from −195.97°Cto 35.06°C within approximate 0.69s upon immersion in the thawing solution. The warming rates reached up to 19, 945 °C/min.

### Recovery and survival rates of frozen oocytes after thawing

**FIGURE 6:**
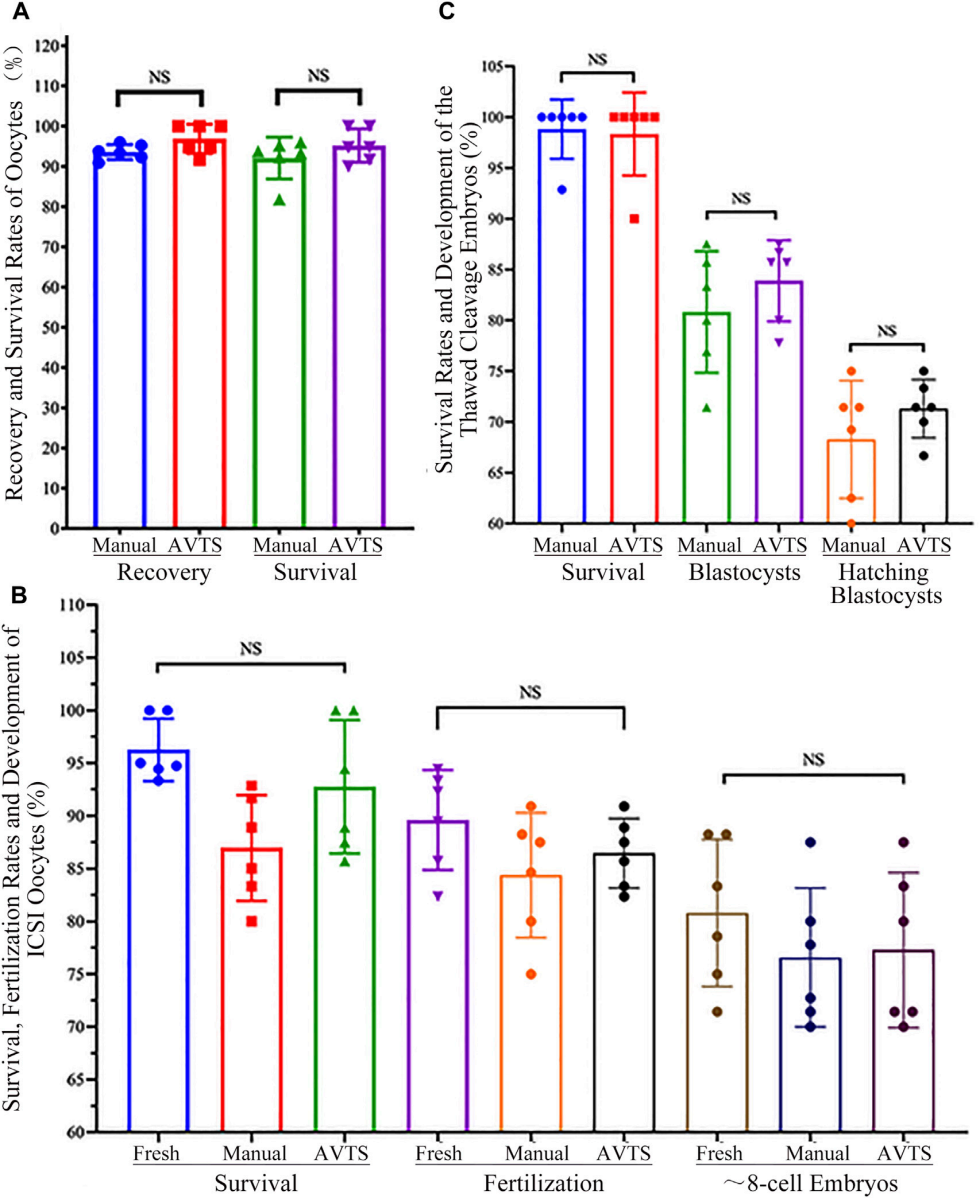
Recovery, Survival and Fertilization Rates of Oocytes/Embryos, and Development of Cleavage Embryo after Vitrification and Thawing. **(A)** Recovery and Survival Rates of Vitrified Oocytes; **(B)** Survival, Fertilization and Cleavage Rates of the ICSI Oocytes; **(C)** Survival Rates and Development of Vitrified Cleavage Embryos. The data on oocyte or embryo recovery, survival rates, oocytes fertilization rates, and the percentages of cleavage embryo or blastocyst were analyzed using one-way ANOVA; the results showed no significant differences (NS) among the groups.

The oocyte survival rates of the manual and AVTS groups were assessed by evaluating their ability to return to a normal physiological volume after thawing, while viability was determined based on the outcomes of oocytes thawing. Statistical analysis revealed no significant differences in the oocyte recovery rates (93.59% ± 1.87% vs. 96.86% ± 3.62%; *p* = 0.654) and survival rates (94.64% ± 4.31% vs. 98.33% ± 4.08%; *p* = 0.699) between the manual and AVTS groups (refer to Figure 6A and Supplementary Table S5).

### Oocyte survival, fertilization rates and embryo development after ICSI

The outcomes of intracytoplasmic sperm injection (ICSI) in the control (fresh oocytes), manual, and AVTS groups (refer to Figure 6B and Supplementary Table S6) indicated that 96.25% ± 2.96%, 86.96% ± 5.02%, and 92.76% ± 6.32% of oocytes were viable, respectively (*p* ≥ 0.214). Among the surviving oocytes, 89.60% ± 4.74%, 84.38% ± 5.91%, and 86.45% ± 3.29% underwent normal fertilization, characterized by the presence of two pronuclei (*p* ≥ 0.988). Moreover, 80.80% ± 6.98%, 76.57% ± 6.59%, and 77.28% ± 7.35% of the fertilized oocytes were able to develop into approximately 8-cell embryos, respectively (*p* > 0.999). Statistical analysis revealed no significant differences in these outcomes among the three groups.

### Development of refrozen cleavage embryos after thawing

Thawed cleavage embryos from both the manual and AVTS groups exhibited 98.81% ± 2.92% and 98.33% ± 4.08% of survival rates (*p* > 0.999). Among the surviving embryos, 80.82% ± 5.98% and 83.90% ± 4.00% in the manual and AVTS groups, respectively (*p* = 0.984), were able to develop into blastocysts by day 3.5. The hatching rates, measured by day 4, were as high as 71.31% ± 2.86% in the manual group and 68.26% ± 5.79% in the AVTS group (*p* = 0.985; Figure 6C and Supplementary Table S7). Notably, no significant differences were observed between the manual and AVTS groups in terms of these developmental outcomes.

## Discussion

Cryopreservation of oocytes is considered a crucial technique for preserving female fertility, particularly for cancer patients undergoing radiation or chemotherapy and for oocyte donation (Huang et al., 2007). Similarly, embryo cryopreservation is essential in traditional IVF treatments to enhance pregnancy rates and ensure patient safety. Furthermore, research on oocyte and embryo cryopreservation in animal models, such as mice, has proven valuable for the preservation of animal resources (Prentice and Anzar, 2010), and may provide preliminary data that could indirectly be applicable to human oocyte and embryo cryopreservation.

So far, vitrification has emerged as a preferred method over slow freezing for oocyte cryopreservation due to its ability to prevent chilling injury and ice crystal formation (Rall and Fahy, 1985; Gupta et al., 2007). This technique has shown high oocyte survival rates and subsequent developmental competence in both animal research and clinical settings (Cobo et al., 2009; Nagy et al., 2009; Nagy et al., 2014; Cobo et al., 2016). The success of vitrification has also led to its application in embryos at various developmental stages (Loutradi et al., 2008; Fasano et al., 2014; Liebermann, 2017). However, there is still room for improvement in terms of the efficiency, consistency, reliability, and safety of vitrification. Therefore, the development of an automated system would be instrumental in standardizing and enhancing the repeatability of vitrification outcomes.

The key features of an automated vitrification system should prioritize the ability to achieve high survival and viability rates for both oocytes and embryos. This can be accomplished by implementing a multi-step process that carefully exposes the cells to increasing and decreasing concentrations of cryoprotectant solutions, thereby minimizing osmotic damage (Arav et al., 1993). Notably, the semi-automated Shara system and the automated Gavi system have demonstrated comparable *in vitro* outcomes in both animal and human studies (Roy et al., 2014b; Roy et al., 2017; Arav et al., 2018; Brunetti et al., 2021). In our study, we have developed a novel carrier cryo-handle (Figure 2) and an automated vitrification and thawing system (Figure 1). This system is designed to be nearly completely operator independent.

During the vitrification process, there is a continuous dynamic change in osmolality caused by cryoprotectant agents (CPAs). At initial equilibrium stage, due to the higher permeability of the cell membrane to water compared to CPAs, the membrane of oocytes or cells undergoes contraction as a result of dehydration. Once the osmolality inside and outside the cells reaches a point of equilibrium, the cell membrane ceases to contract. Subsequently, permeable CPAs and water gradually enter the cells, causing the cells to re-expand.

High concentrations of CPAs and high osmolality play a crucial role in the rapid dehydration process during vitrification. The primary purpose of this dehydration is to minimize the formation of ice crystals inside cells. The osmotic effects can be easily regulated by adjusting the composition of CPAs in the vitrification solutions. However, several studies on oocyte cryopreservation have suggested that the osmotic effects may be more detrimental than the chemical effects (Kono et al., 1991; Shaw et al., 1991; Bautista et al., 1998).

When exposed to high osmolality, oocytes exhibited significant morphological changes during the equilibration and dilution, which might potentially reduce the cell viability (Shaw et al., 1990; Shaw et al., 1991). Therefore, it is crucial to comprehensively understand the oocyte’s tolerance for osmolality in order to improve the efficacy of oocyte cryopreservation. The addition or removal of cryoprotectants during vitrification and thawing should not exceed the oocyte’s tolerance to osmotic changes. A previous study has specifically assessed the oocyte’s tolerance to osmolality induced by commonly used cryoprotectants (Shaw et al., 1991). Furthermore, other studies have found the correlation between the impact on oocyte spindle of cryoprotectants and the occurrence of aneuploidy, as well as the subsequent outcomes of *in vitro* fertilization and embryonic development (Mullen et al., 2004; Huang et al., 2006). A better understanding of the oocyte’s tolerance to osmolality caused by cryoprotectant solutions is crucial for developing an optimal protocol for oocyte cryopreservation. The studies mentioned above clearly highlight the importance of maintaining a suitable transition of osmolality during vitrification and thawing. Therefore, we hypothesize that the viability and developmental potential of oocytes and embryos are similarly influenced by changes in osmolality induced by various cryoprotectant solutions during the automated vitrification and thawing process.

To ensure high survival rates, in addition to exposure time and temperatures, it is crucial to accurately maintain the osmolalities of various VS and TS solutions during vitrification and thawing. Incomplete exchanges between sequential solutions can result in incorrect changes in osmolalities to the cryopreserved cells, which may negatively affect oocyte survival, fertilization, and subsequent embryo development. To demonstrate the ability of AVTS to ensure the stability of osmolalities, we utilized the empty cryo-handle as a simulation tool to measure and compare the differences in osmolalities of collected VS/TS samples with fresh solutions. The results demonstrated that the osmolalities of VS or TS solutions collected from the cryo-handles were almost identical to those of the fresh solutions. Moreover, the change trend of osmolalities was almost completely consistent between fresh and the collected samples (Figure 3), suggesting that all CPAs were fully exchanged during automated vitrification and thawing.

In addition, by employing a cooling rate ranging from 2,500 to 30,000°C/min or higher during vitrification, water undergoes a direct transformation from the liquid phase to a glassy vitrified state (Liebermann, 2012). The bygone belief in the field is that the cooling rate plays a pivotal role in oocyte vitrification and it is preferable to exceed 20,000°C/min (Kuwayama et al., 2005). However, studies conducted on mice have demonstrated that the lethality associated with a slow warming process is attributed to the growth of small intracellular ice crystals through re-crystallization (Seki and Mazur, 2008; Seki and Mazur, 2009), and the warming rate is more critical than the cooling rate (Mazur and Seki, 2011). Interestingly, the authors from the same research team have also shown that a very rapid warming rate, rather than a high cooling rate, is the critical factor for the survival of the oocyte after vitrification and warming (Seki and Mazur, 2012). Furthermore, the data on human or mice subjects also indicated that oocytes can be successfully vitrified with relatively slow cooling and warming rates, while still achieving high rates of survival and successful pregnancies (Cai et al., 2012; Wang et al., 2014). In particular, to achieve extremely high cooling rates was not feasible when closed vitrification devices were utilized. Moreover, it has been shown that the lower cooling rates did not have any detrimental effects on the final outcomes (Liebermann, 2012).

In our study, we conducted a mock experiment to assess the cooling and warming rates of the cryo-handle. Indirect simulation testing is an essential tool for estimating the cooling and warming rates of vitrification devices. This is particularly important as direct measuring methods face challenges in assessing real phenomena within closed spaces, such as closed-type vitrification devices, or confined spaces like micropores with a diameter of less than 1mm, as described in this study. Our results indicate that the cryo-handle exhibits cooling and warming rates of 16,298 and 19,945°C/min respectively, which are within a reasonable range and comparable to those reported for other vitrification devices (Kleinhans et al., 2010; Mazur and Seki, 2011; Arav et al., 2018). Therefore, the cryo-handle has the potential to serve as an alternative device for achieving relatively high cooling and warming rates.

Furthermore, our AVTS demonstrated its ability to automatically control every step of the vitrification and thawing process, including the precise placement of the cryo-carriers and the optimal exposure time to CPAs. Each cryo-handle utilized in the AVTS had the capacity to load four to six oocytes (or embryos). Additionally, during the automated vitrification process, the remaining oocytes or embryos could be conveniently preplaced into the cryo-handles and held in HM for an extended period of time at 37°C. This streamlined approach significantly reduced the total operation time and enhanced the efficiency of both vitrification and thawing procedures.

The results of our oocyte cryopreservation study revealed comparable recovery and survival rates between AVTS and manual procedure (Figure 6A and Supplementary Table S5). These findings strongly suggest that the AVTS is a more suitable option for oocyte cryopreservation.

To prevent the risk of IVF failure resulting from zona pellucida hardening (Johnson et al., 1988), it is essential to perform intracytoplasmic sperm injection (ICSI) when inseminating frozen oocytes. It has been evidenced that the incubation of frozen oocytes in fertilization medium for a duration ranging from 15 min to several hours can effectively reverse cytoskeletal disruption. This practice has been shown to have positive effects on fertilization rates and subsequent embryo development (Eroglu et al., 1998). In our study, it was observed that the thawed oocytes subjected to a 2-h incubation in HTF and subsequently inseminated through ICSI exhibited higher rates of fertilization and subsequent embryo development. Additionally, no significant differences in fertilization and embryo development were found among the control (fresh oocytes), manual and automated vitrification groups (Figure 6B and Supplementary Table S6). Another study demonstrated that the process of vitrification and thawing had minimal impact on the mouse oocyte spindle morphology (Chang et al., 2011). The phase transition and low temperature did not cause significant disruption, enabling the oocyte spindle to promptly recover after thawing. However, we still propose that allowing an adequate recovery period in HTF medium before fertilization could facilitate the reorganization of tubulin and microfilament components of oocytes. This could lead to enhanced fertilization outcomes and improved embryo development. Our results from the AVTS group showed that 86.45% ± 3.29% of the ICSI oocytes that survived were successfully fertilized, and 77.28% ± 7.35% of them developed into approximate 8-cell embryos. These results are comparable to the fertilization and embryo development rates observed in fresh and manually frozen oocytes (Figure 6B and Supplementary Table S6).

In order to further evaluate the efficiency of AVTS, 8-cell cleavage embryos derived from frozen oocyte were re-frozen and thawed using manual and automated procedures. The quality of blastocysts derived was compared between the two procedures. Our results showed that embryos derived from AVTS had similar blastocyst development rates (83.90% ± 4.00% vs. 80.82% ± 5.98%) and similar percentages of hatching blastocysts (71.31% ± 2.86% vs. 68.26% ± 5.79%) compared to the manual vitrification procedure (Figure 6C and Supplementary Table S7). This indicates that the automated system can produce high-quality blastocysts comparable to manual vitrification, making it a suitable alternative for embryo cryopreservation.

This automated system offers several significant advantages, making it a valuable tool in the vitrification and thawing process. Firstly, the oocytes/embryos are contained within a single cryo-carrier throughout the entire procedure, from the initial step of exposure in HM to the final step of being plunged into LN. This eliminates the need for multiple steps of oocyte/embryo observation and transfer under a stereo-microscope, thereby reducing the workload for operators. Additionally, the bio-sample movements in different solutions can be controlled in an automated and precisely timed manner, thereby guaranteeing the accuracy and consistency of the vitrification operation and reducing the influence from human factor. Furthermore, the system also incorporates an automated thawing function, thereby achieving full automation of both the vitrification and thawing processes. Therefore, this automated system is highly beneficial in liberating operators from time-consuming and tedious routine tasks, and is worthy of widespread adoption for oocyte or embryo cryopreservation.

## Conclusion

In summary, the automated vitrification and thawing system does not affect the osmolality of VS or TS solutions, ensuring the proper exchange of cryoprotectants during vitrification and thawing. Moreover, the cryo-handle facilitates rapid cooling and warming rates, which are advantageous for oocyte and embryo cryopreservation. Furthermore, the findings from mouse oocytes and embryos prove that automated system offers significant advantages in terms of maintaining oocyte survival, promoting successful fertilization, and supporting subsequent embryonic development. Therefore, the automated vitrification and thawing system is inevitably becoming a superior alternative to traditional manual operation.

## Data Availability

The original contributions presented in the study are included in the article/Supplementary Material, further inquiries can be directed to the corresponding author.
