# Correction: Automation in vitrification and thawing of mouse oocytes and embryos

**DOI:** 10.3389/fcell.2025.1535951

**Published:** 2025-07-23

**Authors:** Yan Zhu, Quan-Jun Zhang, Huai L. Feng, Jin Luo, Shu Miao, Man-Xi Jiang

**Affiliations:** 1 Guangdong Second Provincial General Hospital, Guangzhou, China; 2 Institutes of Biomedicine and Health, Chinese Academy of Sciences, Guangzhou, China; 3 New York Fertility Center, New York-Prebyterian Healthcare System Affiliate Weill Cornell Medical College, New York, NY, United States; 4 T Stone Robotics Institute, Department of Mechanical and Automation Engineering, The Chinese University of Hong Kong, Shenzen, China; 5 Department of Automation, Tsinghua University, Beijing, China

**Keywords:** cryo-handle, automated vitrification and thawing, oocytes, embryos, mice

In the published article, there was an error in the approval number of the ethical committee of Guangdong Second Provincial General Hospital for animal experiments.

A correction has been made to **Materials and Methods**, *Humane Care and Use of Animals*, Paragraph 1. This sentence previously stated:

“All experimental protocols were performed after obtaining the approval from the ethical committee of Guangdong Second Provincial General Hospital for animal experiments (No. 2014-KYLLM-065) and all the used methods are reported in accordance with the Animal Research Reporting of *in vivo* Experiments (ARRIVE) guidelines.”

The corrected sentence appears below:

“All experimental protocols were performed after obtaining the approval from the ethical committee of Guangdong Second Provincial General Hospital for animal experiments (No. 2014-KYLL-015) and all the used methods are reported in accordance with the Animal Research Reporting of *in vivo* Experiments (ARRIVE) guidelines.”

The authors apologize for this error and state that this does not change the scientific conclusions of the article in any way. The original article has been updated.

